# Layered host–guest long-afterglow ultrathin nanosheets: high-efficiency phosphorescence energy transfer at 2D confined interface†
†Electronic supplementary information (ESI) available. See DOI: 10.1039/c6sc03515a



**DOI:** 10.1039/c6sc03515a

**Published:** 2016-09-06

**Authors:** Rui Gao, Dongpeng Yan

**Affiliations:** a State Key Laboratory of Chemical Resource Engineering , Beijing University of Chemical Technology , Beijing 100029 , P. R. China . Email: yandp@mail.buct.edu.cn; b Beijing Key Laboratory of Energy Conversion and Storage Materials , College of Chemistry , Beijing Normal University , Beijing 100875 , P. R. China . Email: yandp@bnu.edu.cn

## Abstract

Tuning and optimizing the efficiency of light energy transfer play an important role in meeting modern challenges of minimizing energy loss and developing high-performance optoelectronic materials. However, attempts to fabricate systems giving highly efficient energy transfer between luminescent donor and acceptor have achieved limited success to date. Herein, we present a strategy towards phosphorescence energy transfer at a 2D orderly crystalline interface. We first show that new ultrathin nanosheet materials giving long-afterglow luminescence can be obtained by assembling aromatic guests into a layered double hydroxide host. Furthermore, we demonstrate that co-assembly of these long-lived energy donors with an energy acceptor in the same host generates an ordered arrangement of phosphorescent donor–acceptor pairs spatially confined within the 2D nanogallery, which affords energy transfer efficiency as high as 99.7%. Therefore, this work offers an alternative route to develop new types of long-afterglow nanohybrids and efficient light transfer systems with potential energy, illumination and sensor applications.

## Abstract 2

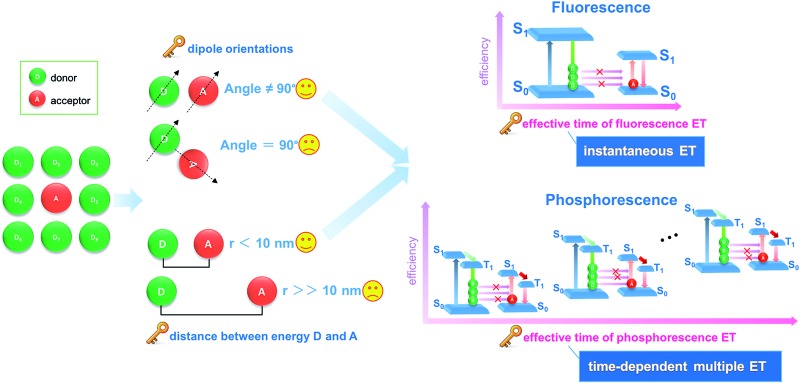
The orderly assembly of photoactive donor and acceptor phosphors within the gallery of 2D layered nanosheets presents obviously long-lived luminescence and effective energy transfer.

## Introduction

**Fig. 1:**
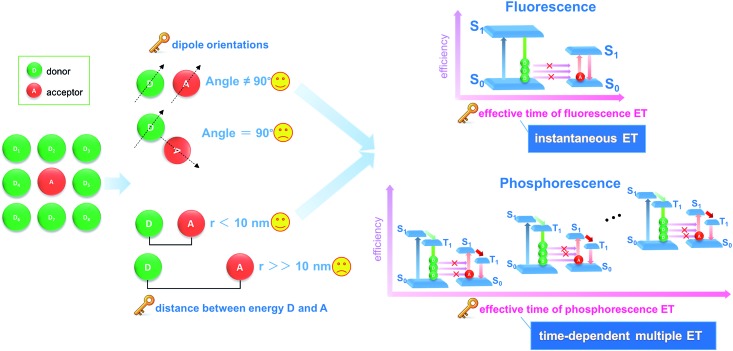
Schematic illustration of different energy transfer (ET) processes showing how phosphorescence energy transfer (PET) leads to more efficiency than conventional fluorescence energy transfer (FET).

Energy transfer and interchange are common phenomena in nature,^1,2^ and are also becoming increasingly important in the energy,^3,4^ materials^5,6^ and environmental fields.^7^ From both academic and engineering perspectives, improvement in energy transfer efficiency plays a key role in saving energy and reducing our impact on the environment.^8–11^ Recently, the transfer of light energy has attracted extensive attention, since light offers the fastest transport speed and can also be recognized and monitored easily. For example, the molecule-based fluorescence energy transfer (FET) technology has been used effectively in molecular biology^12–15^ and optoelectronic applications^16^ (such as optical probes,^17^ information communication,^18^ and light-emitting diodes^19^). However, increasing the efficiency of light energy transfer in a typical FET process remains a considerable challenge, since the rapid release of energy from a photoactive donor (D, excited state lifetime: 10^–9^ to 10^–8^ s) results in an instantaneously high energy density, which cannot be effectively harvested by the acceptor (A) molecules (Fig. 1).^20,21^ Additionally, from both energy and spatial points of view, the D–A pairs should also meet the requirements of matched energy levels, close distance (<10 nm), and suitable orientation in order for the energy transfer process to be successful.^21,22^

Materials with afterglow luminescence (*e.g.* persistent phosphorescence), which can last for an appreciable time after removal of the excitation source, have aroused particular interest during the last several decades by virtue of their long-lived excited states and prolonged emission times.^23–26^ Such afterglow materials can release light energy much more slowly compared with typical fluorescent systems, and thus offer energy-storage light-emitting properties.^27,28^ Therefore, in principle, afterglow materials could serve as an effective energy donor D, because instead of the instantaneous spike in energy density associated with an FET donor D, energy is released and averaged on a longer time scale which should lead to a full energy transfer between D and A units (Fig. 1). However, to date, an efficient phosphorescence energy transfer (PET) system has still very rarely been explored, probably because the long-lived emissive route of D is not easily available with the appearance of an A unit, since the long-lived excitation generally involves the conversion of the spin-allowed short-lived singlet states to the spin-forbidden triplet states. Studies of afterglow materials have mainly focused on transition metal or rare-earth (RE)-containing inorganic materials;^29^ however, their high cost and relatively complicated synthesis methods (*e.g.*, high-temperature solid-state process) have restricted their practical applications. In recent years it has been reported that certain organic compounds, mainly containing carbonyl or carboxylate groups, can exhibit room temperature phosphorescence (RTP) in the crystalline state although no phosphorescence is observed when the materials are amorphous or in solution.^30–33^ This has been explained in terms of the intermolecular interactions in the ordered crystal being able to restrict intramolecular motions to such an extent that the normally rapid nonradiative loss of triplet excitons is significantly inhibited. For example, Kim *et al.* have observed that purely organic crystals containing heavy atoms and carbonyl groups can present RTP with quantum yields of 0.5–2.9%, and the values were obviously enhanced upon formation of mixed crystals.^30*a*^ Tang *et al.* have shown a series of aromatic phthalic acid analogues with crystallization-induced RTP enhancement.^31*a*^ Jin *et al.* have illustrated that the formation of cocrystal can be a potential way to enhance the RTP quantum yield.^32^ However, it may be difficult to obtain large quantities of suitable single crystals for practical applications.

**Fig. 2:**
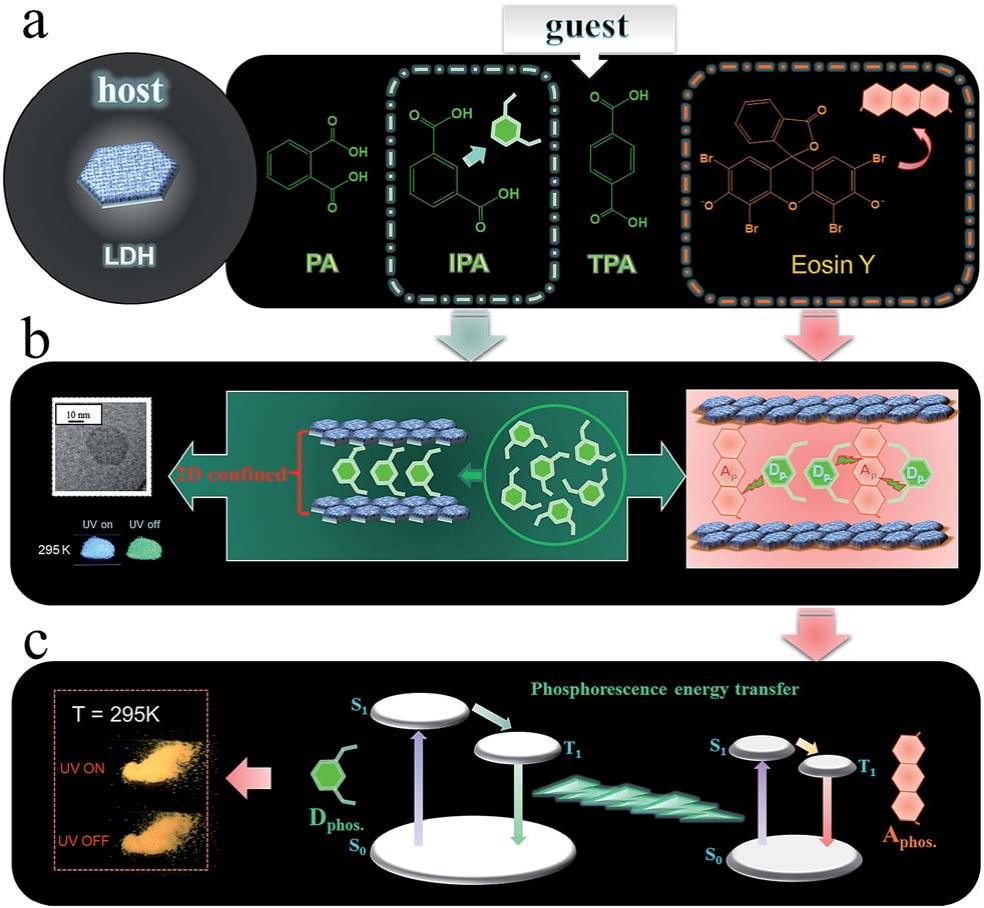
Schematic representation of the LDH host and D/A guest species (a, b) and the proposed mechanism for PET (c).

Herein, we have put forward a strategy to achieve a potential PET process by incorporating both the donor D and the acceptor A in the interlayer nanogallery of a layered inorganic host material. Firstly, the design involves selecting possible D and A phosphor units with their photoluminescence (PL) located at blue-green and orange-red regions, respectively (Fig. 2a), in which the absorption band of A overlaps strongly with the phosphorescence band of D, but has minimal overlap with the fluorescence band of D. This feature facilitates the construction of a PET route rather than the common FET. Secondly, the restrictions on the packing of co-accommodated D and A guests at a 2D confined interface should be less severe than in a highly crystalline material, which may allow the adoption of a suitable orientation for effective energy transfer. The layered low-dimension host nanochannel can also concentrate the uniform energy transfer direction and restrain the light energy loss.^34,35^ To test this hypothesis, graphene-like layered double hydroxide (LDH) was selected as the 2D host material to accommodate the D/A guests. LDH presents a large family of layered anionic clays which can be described by the general formula [M^II^_1–*x*_ M^III^_*x*_(OH)_2_]^*z*+^A^*n*–^_*z*/*n*_·*y*H_2_O (M^II^ and M^III^ are divalent and trivalent metal ions, respectively; A^*n*–^ is an anion).^36^ The structure of the LDH layer is based on brucite with edge-sharing M(OH)_6_ octahedra, and partial substitution of M^2+^ cations by M^3+^ inducing a positive charge on the host layers, which can be further balanced by anions in the hydrated interlayer galleries. In contrast to most cationic clays (where the layers have a negative charge), the charge density and elemental composition of LDH can be varied over a wide range in the synthesis process, which facilitates fine control of the properties of the host layers.^37^ It is known that the rigid and crystalline LDH sheets provide a nano-scaled anisotropic microenvironment for guest chromophores,^38–40^ which favors the formation of an orderly arrangement of phosphor aggregate states with prolonged excitation lifetime. Moreover, the positively charged LDH layers can supply an interlayer electric field for the polarization of guest molecules, and thus should accelerate the dipole–dipole interactions between D and A.^36,41–43^

As the potential donor species D, we chose three isomeric benzene dicarboxylates because the n → π* transition of the carboxylic group is favorable for the enhancement of spin–orbit coupling and long-lived triplet excited states after being fixed into the 2D nanogallery of LDH.^31^ The resulting host–guest nanohybrid materials exhibited long-lived green RTP (Fig. 2a, afterglow time range: 0–6 seconds), showing that formation of 2D intercalation nanohybrids is indeed a feasible alternative to crystallization as a means of increasing the phosphorescence lifetimes of such simple organic species. Interestingly, the phosphorescence shows a reversible temperature-stimulus response. Then, Eosin Y was selected as the A guest, since it is known that the heavy atom effect of its four Br substituents enhances the spin–orbital coupling^44^ and its absorption band overlaps extensively with the afterglow emission from the LDH–benzene carboxylate host–guest materials. Co-intercalation of benzene dicarboxylate and Eosin Y guests with different molar ratios in LDH hosts afforded (D@A)/LDH materials showing effective PET, with efficiency up to 99.7%, and the lifetime of the orange-red phosphorescence of interlayer Eosin Y (A) being significantly increased (Fig. 2b and c). To the best of our knowledge, this work involves the first proof of the concept of combining both host–guest long-lived afterglow nanohybrids and high-efficiency PET at a 2D confined interface. Therefore, this work not only paves an effective way to develop inexpensive (noble-metal-free and rare-earth-free) long-afterglow phosphors, but also supplies an alternative strategy to obtain emerging layered D/A nanohybrids towards high-efficiency light energy transfer.

## Results and discussion

### Structure and morphology of host–guest afterglow nanohybrids

**Fig. 3:**
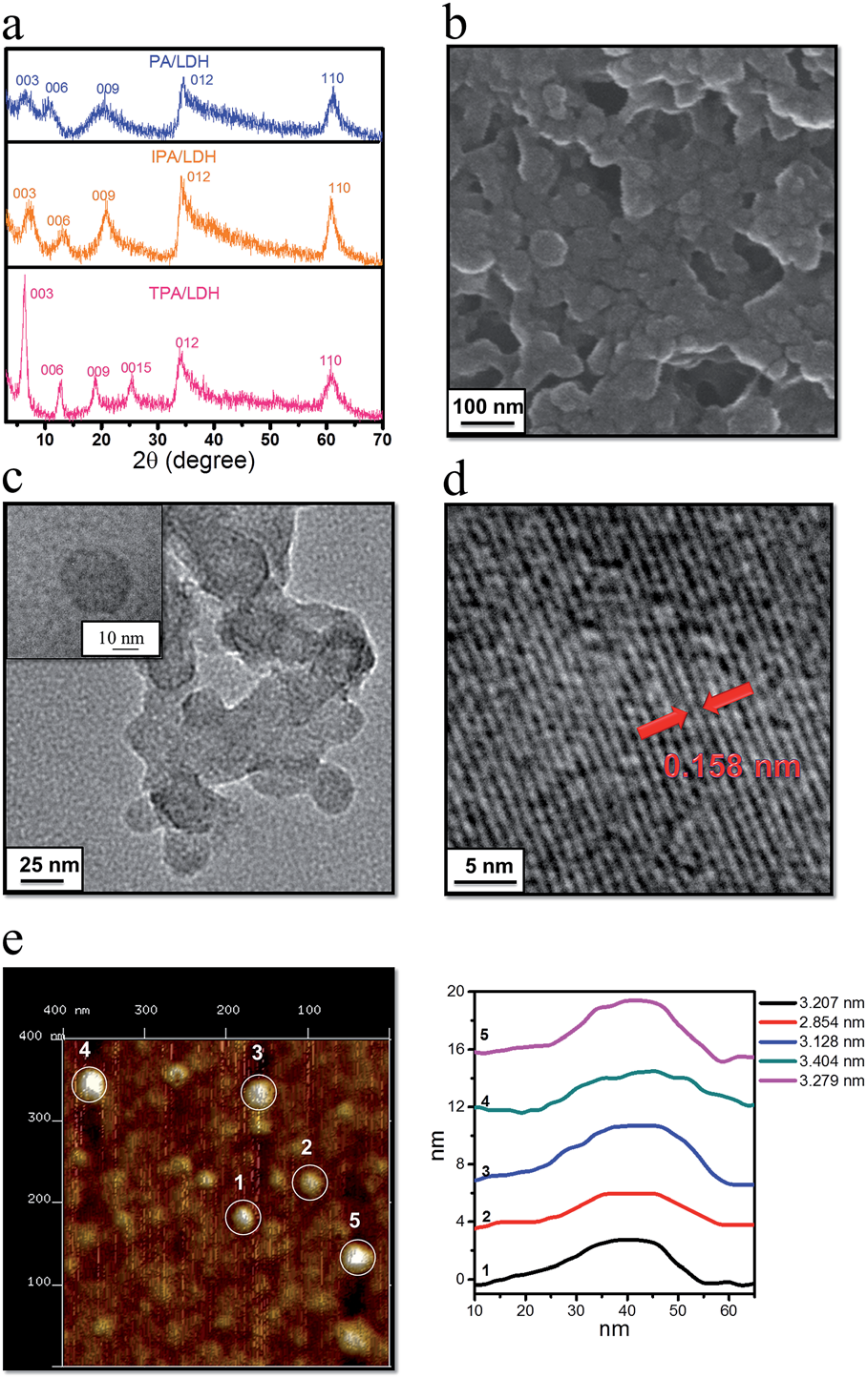
Structure and morphology of LDHs nanosheets. (a) XRD patterns for PA/LDH, IPA/LDH and TPA/LDH. (b) SEM image for IPA/LDH. (c) The TEM image and (d) high-resolution TEM of IPA/LDH (inset image in c shows an individual IPA/LDH nanosheet). (e) AFM image and the height curves for IPA/LDH.

Three benzene dicarboxylic acid isomers (namely phthalic acid (PA), isophthalic acid (IPA), and terephthalic acid (TPA)) were assembled into the interlayer gallery of a Zn–Al-LDH host using a co-precipitation method. Powder X-ray diffraction (PXRD, Fig. 3a) patterns show that, in each case, all the reflections for the samples can be indexed to a rhombohedral lattice with *R*3*m* symmetry, which is commonly used for the description of the 3*R*-type LDH structure. Taking IPA/LDH as the example (which has the highest emission lifetime as shown below), the main characteristic reflections appear at 7.26° (003), 13.38° (006), 20.94° (009), and 60.74° (110), respectively. Values of *d*_003_ (1.220 nm), *d*_006_ (0.666 nm) and *d*_009_ (0.431 nm) present a good multiple relationship between the basal, second and third-order reflections. The lattice parameter *c* can be calculated by averaging the positions of the three harmonics: *c* = 1/3(*d*_003_ + 2*d*_006_ + 3*d*_009_) = 1.282 nm. The interlayer spacings for PA/LDH and TPA/LDH (1.463 nm and 1.414 nm) are slightly larger than that of IPA/LDH, respectively, suggesting that the guest species adopt different interlayer packing arrangements in the LDH nanogallery. Elemental analysis results (ESI Table S1†) show that the Zn^2+^/Al^3+^ molar ratios in the products are close to the nominal ones, suggesting ordered structures for the Zn–Al-LDH layers. Solid-state NMR is a powerful method for characterizing inorganic–organic hybrids and understanding host–guest interactions and molecular packing. The experimental ^13^C MAS NMR spectra of the pristine PA, IPA, TPA and their hybrid composites are shown in ESI Fig. S1.† For the pristine IPA sample (ESI Fig. S1b†), the experimental NMR peaks are consistent with the simulated ones. The peak at 129.83 ppm is assigned to the sp^2^ carbon atoms (C_1_, C_3_ and C_5_) within the phenyl ring, and it moved to 133.10 ppm in the intercalated product. The sp^2^ carbon atoms (C_2_) in the carboxyl shifted from 172.46 ppm to 173.74 ppm upon intercalation. Moreover, a weak peak at 145.9 ppm of the sp^2^ carbon atoms (C_5_) in the pure IPA disappeared in the IPA/LDH, and has been replaced by the strong peak at 133.10 ppm. Therefore, it can be concluded that most of the resonances of the sp^2^ carbon in the interlayer IPA underwent a shift downfield, suggesting that the positively-charged LDH layer can polarize and delocalize the electronic density of the anionic IPA to some extent. We also found a similar trend in both PA/LDH and TPA/LDH intercalation products. Thermogravimetric analysis further revealed that the decomposition temperature of IPA/LDH appeared at 490 °C, which is higher than that of pure IPA, suggesting that the thermal stability of the IPA improved upon intercalation (ESI Fig. S2†).

To probe the morphological features of intercalated LDHs, scanning electron microscopy (SEM) was performed on the samples. It was observed that the LDH surface is microscopically uniform (Fig. 3b and ESI Fig. S3†). Furthermore, transmission electron microscopy (TEM, Fig. 3c) revealed that IPA/LDH consisted of pseudo-hexagonal nanosheets with a diameter distribution of *ca.* 25–40 nm. High-resolution TEM (Fig. 3d) showed well-defined crystal planes with an average lattice distance of *ca.* 0.158 nm, corresponding to the (110) direction of the LDH monolayer, which also suggests a high degree of order within the LDH nanosheets. To investigate the surface microstructure and thickness of the as-prepared sheet-like LDHs, the inorganic–organic hybrids were further monitored by atomic force microscopy (AFM). The typical tapping mode AFM image for IPA/LDH nanohybrids (Fig. 3e) shows that the thickness values of the LDHs can be estimated as *ca.* 3–4 nm, confirming the formation of the IPA/LDH ultrathin nanosheets.

### Host–guest layered nanosheets with afterglow luminescence

**Fig. 4:**
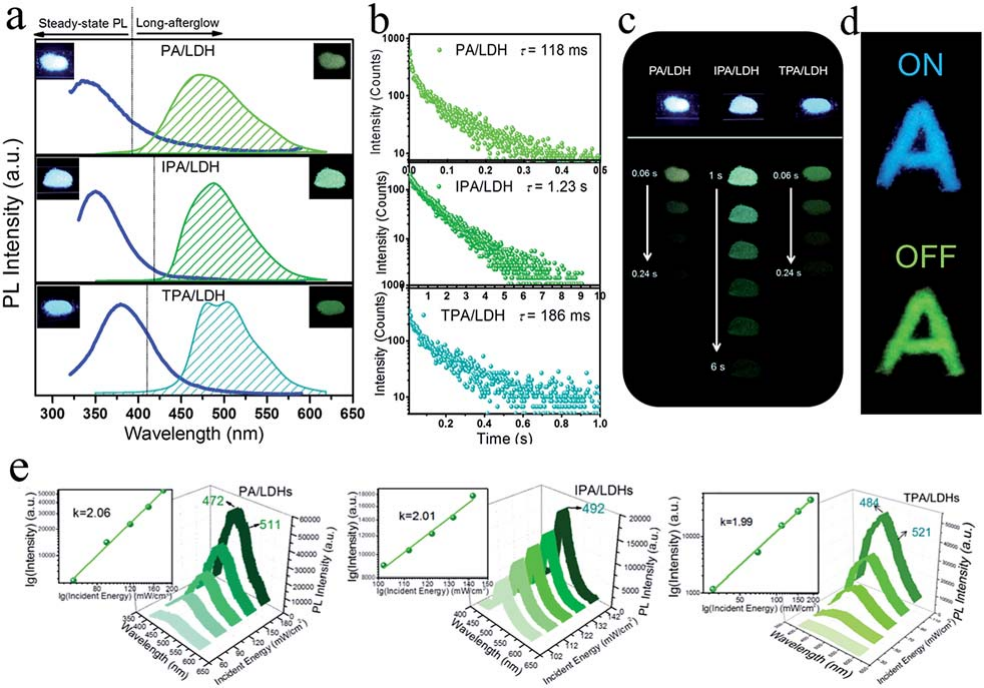
(a) Steady-state fluorescence (left) and phosphorescence (right) spectra of PA/LDH, IPA/LDH and TPA/LDH nanohybrids. Insets show the corresponding photographs taken before (left) and after (right) the excitation source is turned off. (b) Time-resolved emission decay profiles of PA/LDH (at 475 nm), IPA/LDH (at 489 nm), and TPA/LDH (at 480 nm). All compounds were excited at 320 nm. (c) Photographs of the long-afterglow materials under ambient conditions taken at different time intervals before and after turn-off of the excitation. (d) The letter ‘A’ made with ultralong phosphorescent IPA/LDH can be unmistakably identified by the naked eye after the excitation is switched off. (e) Photoemission spectra of PA/LDH, IPA/LDH and TPA/LDH excited by an 800 nm laser with different incident powers.

On UV irradiation at 320 nm, intense blue emissions (*λ* ^em^: 336, 351 and 380 nm) were observed from the PA/LDH, IPA/LDH and TPA/LDH nanohybrids (Fig. 4a). Compared with the pristine PA, IPA and TPA, the emission wavelengths all had a blue shift upon intercalation (ESI Fig. S4†), which can be attributed to the formation of an H-type aggregation in the 2D confined region.^41^ Interestingly, upon removal of the UV source, the emission changed to green in color (Fig. 4a) and slowly faded, as could be easily recognized by the naked eye. Time-resolved luminescence decay showed that the three LDH systems all exhibited relatively long phosphorescence lifetimes (0.118, 1.23 and 0.186 s, Fig. 4b), and all detailed excited state lifetime values are summarized in Table S2 (ESI†). The corresponding RTP quantum yields of PA/LDH, IPA/LDH and TPA/LDH are 0.71%, 3.02% and 0.69%, respectively. These values are comparable to those as-reported powdered phosphorescent systems^30–33^ as tabulated in Table S3 (ESI†). As a result of the high photoemission lifetime of the IPA/LDH nanosheets, afterglow emission at room temperature can be detected as long as 6 seconds after removal of the UV excitation (Fig. 4c). Such long-persistent emission decay is significantly higher than those recently reported for free PA (0.5 ms) and free IPA (0.290 s) in the single crystal states,^31^ and also higher than for most current rare-earth-free molecule-based and metal-complex-based afterglow systems.^33^

Furthermore, to illustrate the potential use of LDH-based materials as an anti-counterfeiting tool, the letter “A” was made by IPA/LDH (Fig. 4d). Upon irradiation with a 320 nm UV lamp, a bright blue letter “A” can be observed. The color of the letter “A” turned to green and could still be observed by the naked eye after the UV lamp was turned off. Such a change in emission from blue to green is totally different from the behavior of typical rare-earth-containing afterglow phosphors, which show the same emission color before and after removal of the UV source. This behavior may make the LDH nanosheets useful as optical antiforgery materials for information security applications. In addition, the influence of oxygen on the phosphorescence intensity was further investigated. The phosphorescence intensity decreased only slightly in air compared with that under nitrogen conditions. However, the intensity decreased markedly under an oxygen atmosphere, as is typically observed for phosphorescent materials, since the triplet excited states are sensitive to oxygen (ESI Fig. S5†).

Besides the down-conversion long-afterglow properties, we found that the phosphorescence could also be available through an up-conversion process. Upon excitation by an 800 nm laser light, the PA/LDH, IPA/LDH and TPA/LDH systems exhibited obvious low-wavelength phosphorescence, with the emission peaks close to those excited by irradiation at 320 nm, as discussed above (Fig. 4e). Moreover, plots of log(intensity) *vs.* log(incident energy) showed a good linear relationship, with the slopes close to 2, suggesting that the up-conversion process involves a two-photon mechanism. As far as we know, materials exhibiting such one/two-photon long-afterglow emission have not previously been reported in an LDH-based luminescent system, and thus this work may also open up a broad class of layered host–guest afterglow nanohybrids for illumination and light-emitting applications.

### Theoretical studies on the IPA/LDH

**Fig. 5:**
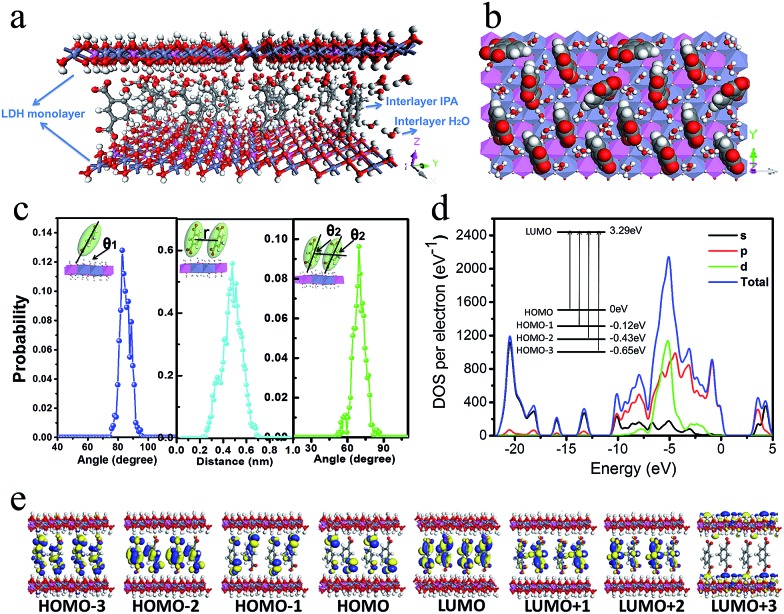
(a) and (b) Typical snapshots of MD simulation for IPA between the Zn–Al-LDH monolayers. (c) The distributions of orientational angle *θ*_1_ (the plane of IPA with respect to the LDH monolayer) (left). The distribution of the central distance between two nearest neighboring IPA anions for the IPA/LDH system (middle). The distribution of orientational angle *θ*_2_ between transition dipole vector of IPA and the lineation vector of two nearest IPA anion centers (right). (d) Total and partial electronic density of state (TDOS and PDOS) and (e) Frontier orbital profiles for the IPA/LDH system.

To better understand the orientation and aggregation states of the IPA within Zn–Al-LDH nanosheets, molecular dynamic (MD) simulations were performed on an idealized IPA/LDH model (*T* = 293 K, *P* = 0.1 MPa). It was observed that the most probable angle of IPA relative to the LDH monolayer appears at *ca.* 83° (Fig. 5c, left), suggesting that the IPA anions present a single-layer stacking fashion, and are nearly perpendicular to the LDH layers. Typical side-view and top-view snapshots capturing the configuration of IPA/LDH nanosheets are shown in Fig. 5a and b. Additionally, the average center–center distance (Fig. 5c middle) between adjacent IPA anions is *ca.* 0.48 nm, which facilitates the formation of strong π–π interactions between the interlayer phosphors. The relative orientation (between the dipole vector of IPA and the center of the lineation vector) of two neighboring IPA anions is mainly populated in the range from 51 to 86° (Fig. 5c, right) with an optimal angle of 69°, indicating the formation of H-type IPA dimers between LDH nanosheets. Such stacking fashion is also in agreement with the luminescent blue-shift of the IPA/LDH compared with pure IPA. Recently, Huang *et al.* have proposed that the construction of H-aggregates can stabilize the lowest excited triplet states, and prolong the RTP lifetime.^33*a*^ Therefore, in this work, the occurrence of H-type IPA dimers in the 2D nanosheets may correspond to the enhancement of the phosphorescence emission.^41^

To obtain information about the electronic structure and excited state of the IPA/LDH 2D hybrid nanosheets, a periodic time-dependent density functional theoretical (TDDFT) calculation was further carried out. Total and partial electronic densities of states (TDOS and PDOS) reveal that the top of the valence band and the bottom of the conducting band of the IPA/LDH system are mainly dominated by the p orbitals from C/O atoms in the IPA units (Fig. 5d and ESI Fig. S6†). Near the Fermi level, the 2p electrons of O atoms from IPA make the main contribution to the TDOS. The band structure profile shows that IPA/LDH has a small band gap of 3.3 eV, which may correspond to the fluorescence observed experimentally at 351 nm (ESI Fig. S7†). The energy bands around the Fermi level are almost independent of the *k* electron wave vectors along the GZ line ([001] direction), indicating the strong electron confinement effect of the host layer on IPA in the normal direction. The obvious dependence of *k* on band energy along GF (the direction of the two interlayer IPA) also indicates the occurrence of electron dispersion and confirms the interactions between adjacent IPA anions in the LDH gallery, corresponding to the H-type dimers as described in the MD simulation.

Frontier orbital analysis (Fig. 5e) further shows that the highest occupied molecular orbitals (HOMOs) and the lowest unoccupied molecular orbitals (LUMOs) are mainly derived from the C/O atomic orbitals in the π-conjugated benzene and carboxylic groups of IPA. The four lowest effective excitations are mainly from the HOMO–*n* (*n* = 0, 1, 2, 3) → LUMO, corresponding to π → π* and n → π* transitions for the fluorescence and phosphorescence processes, respectively. Moreover, it was observed that the Zn–Al-LDH monolayer contributed to the LUMO+3, but does not participate in the photoexcitation process. The results demonstrate that the fluorescence and phosphorescence both derive from IPA, and no electronic and energy transfer occurs between IPA and LDH nanosheets, and thus no energy loss is observed in the *c*-axis direction. The theoretical calculations thus suggest that the light energy transport can be highly confined within the 2D LDH interfaces.

### Reversible temperature-responsive phosphorescence

**Fig. 6:**
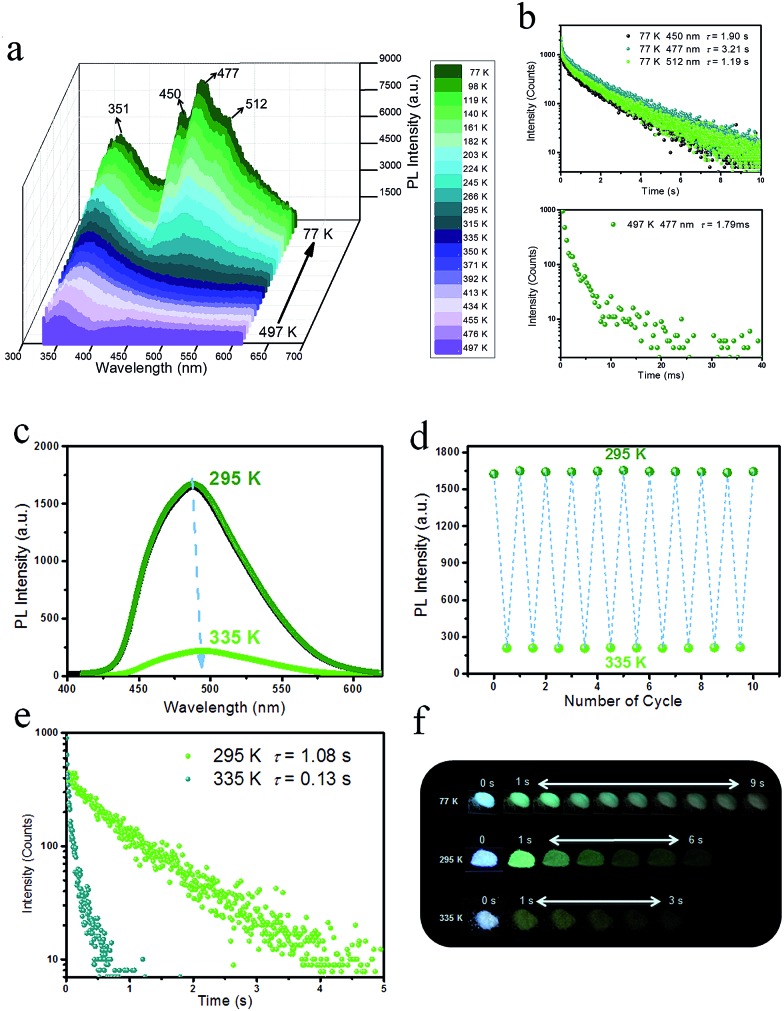
(a) Steady-state PL spectra of IPA/LDH recorded with increasing temperatures from 77 to 497 K. (b) Lifetime decay profiles at 77 K and 497 K. (c) Steady-state PL spectra of IPA/LDH recorded with increasing temperatures from 295 to 335 K. (d) Heating–cooling cycles of the PL intensity. (e) Lifetime decay profiles at temperatures of 295 K and 335 K. (f) Photographs of the long-afterglow nanosheets taken at different time intervals before and after turn-off of the excitation at different temperatures.

To detect the potential heat-related afterglow luminescence of 2D layered hybrid nanosheets, temperature-dependent emission spectra of the IPA/LDH were also measured. On decreasing the temperature from 295 to 77 K, the emission intensity of IPA/LDH increased obviously (Fig. 6a), and the three phosphorescence bands at 450 nm, 477 nm and 512 nm became more prominent (Fig. 6a). The isolated bands can be attributed to different triplet excitation states (T_1_, T_2_ and T_3_) of IPA/LDH nanosheets. Moreover, the time-resolved emission decay was also prolonged at low-temperature, and the afterglow can be traced in the 0–9 second range (Fig. 6b and f); while on increasing the temperature from 295 to 497 K, the PL intensity at 477 nm decreased dramatically relative to that at 351 nm (Fig. 6a), and the phosphorescence lifetime also decreased with a value of 1.79 ms at 497 K. Such behavior is expected as the energy of molecular vibrations and consequent nonradiative loss increased with increasing temperature. Additionally, by recycling the sample between temperatures at 295 and 335 K, it was observed that the changes in phosphorescence emissive intensity can be readily repeated at least 10 times during heating–cooling cycles (Fig. 6c and d), suggesting that the IPA/LDH nanosheets could serve as a temperature-responsive phosphorescence switch by virtue of these reversible changes in emission.

### Phosphorescence energy transfer of the afterglow nanosheets

**Fig. 7:**
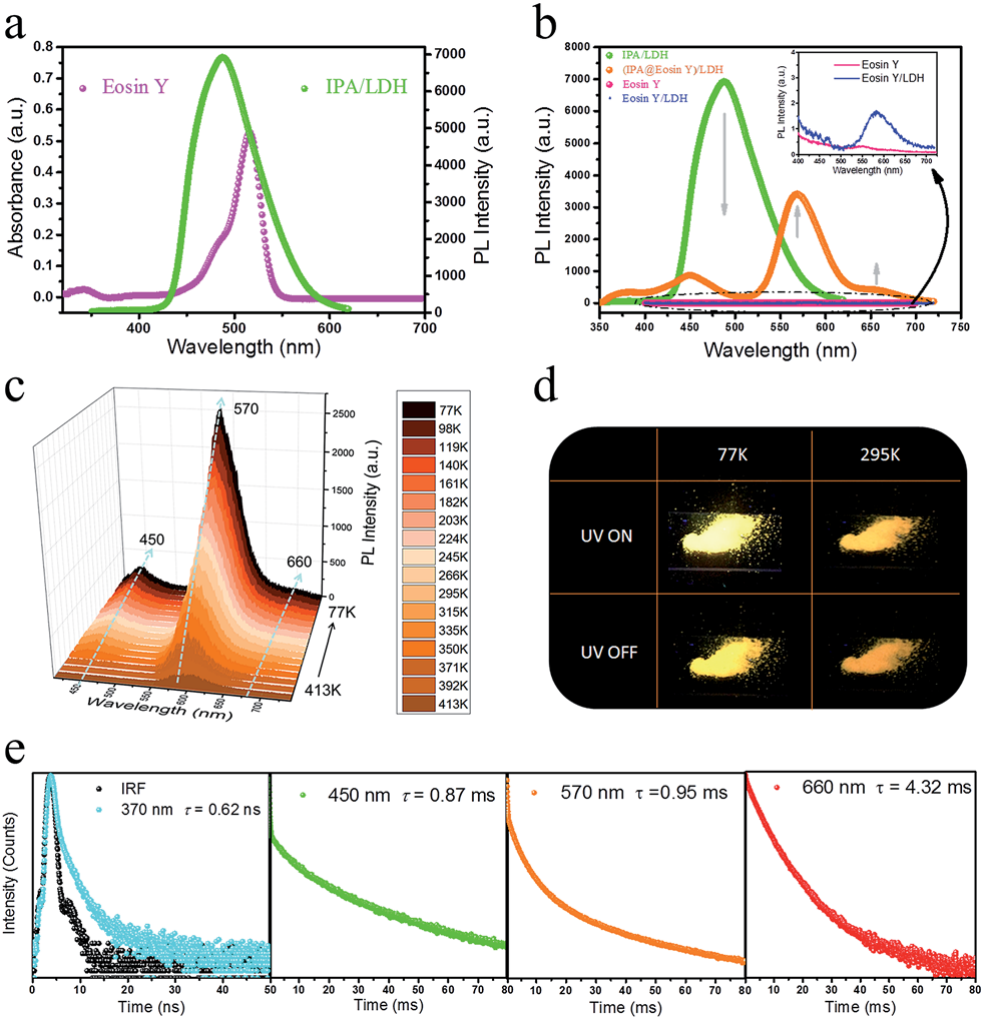
(a) UV-Vis absorption spectrum of pure Eosin Y and phosphorescence spectrum of IPA/LDH. (b) Phosphorescence spectra of pure Eosin Y, IPA/LDH, Eosin Y/LDH and (IPA@Eosin Y)/LDH. (c) Steady-state PL spectra of (IPA@Eosin Y)/LDH recorded at decreasing temperatures from 413 to 77 K. (d) Photographs of (IPA@Eosin Y)/LDH at 77 K and 295 K before and after turn-off the excitation. (e) Lifetime decay profiles of (IPA@Eosin Y)/LDH at 295 K monitored at 370, 450, 570 and 660 nm.

Having established that intercalation in 2D LDH nanosheets is an effective alternative to crystallization as a way of restricting the intramolecular motion of IPA and thus generating long-afterglow emission, the final step is to investigate whether the system can act as a PET donor D by taking advantage of the ability to co-intercalate a second guest species in the LDH host which can act as a PET acceptor. Eosin Y was chosen as the potential energy transfer acceptor A, due to the high degree of overlap between the afterglow emission from IPA and absorption from Eosin Y in the range 460–545 nm (Fig. 7a). Furthermore, its negative charges should allow Eosin Y to be co-intercalated into the LDH nanogallery and thus afford close contact between the D–A pair. IPA and Eosin Y with different ratios (90 : 10–100 : 1) were co-assembled into the LDH host using a co-precipitation method. PXRD shows that there is no obvious change in the interlayer spacing compared with the pristine IPA/LDH system, and the crystalline characteristics confirm the ordered arrangement of the two guest species between the LDH layers (ESI Fig. S8a†). SEM shows that the (IPA@Eosin Y)/LDH has retained the same nanosheet-like morphology (ESI Fig. S9†) as IPA/LDH. Phosphorescence spectra of (IPA@Eosin Y)/LDH samples with different ratios (90 : 10–100 : 1) show that the highest phosphorescence intensity occurs for the (IPA@Eosin Y)/LDH with a D : A ratio of 100 : 1 (ESI Fig. S8b†).

Compared with the pristine IPA/LDH, the phosphorescence emission in the range 425–520 nm for (IPA@Eosin Y)/LDH decreased dramatically upon excitation at 320 nm. Moreover, the phosphorescence band located at 450 nm became more prominent relative to those at 477 and 512 nm due to the high absorption of Eosin Y in the range 460–545 nm (Fig. 7b). Compared with the pristine Eosin Y/LDH system, the luminescence of (IPA@Eosin Y)/LDH shows an obvious increase, in which the *λ* ^em^ at 570 nm and a shoulder peak at 660 nm can be assigned to the decay fluorescence and triplet phosphorescence, respectively (Fig. 7b and ESI Fig. S10a†). Besides the decrease in the PL intensity from IPA and the increase in the PL intensity of Eosin Y, the lifetime of the IPA (D) at 450 nm has significantly reduced from 262 ms to 0.87 ms (Fig. 7e and ESI Fig. S11b†), whilst that belonging to the Eosin Y (A) increased from 0.12 ms to 0.95 ms (Fig. 7e and ESI Fig. S10b†). The emission quantum yield value of the (IPA@Eosin Y)/LDH is 2.67%, which is higher than that of (Eosin Y)/LDH (2.05%). These observations clearly confirm the occurrence of PET in the (IPA@Eosin Y)/LDH nanohybrid. Moreover, the PL lifetime of (IPA@Eosin Y)/LDH appearing at *ca.* 370 nm is almost identical to that of the pristine IPA/LDH nanosheets (Fig. 7e and ESI Fig. S11a†), suggesting the absence of FET during the photoemission process. The PET efficiency (*E*_P_) of the (IPA@Eosin Y)/LDH can be estimated quantitatively according to the equation *E*_P_ = 1 – *τ*_DA_/*τ*_D_, where *τ*_DA_ and *τ*_D_ are the PL lifetime values of D in the presence and absence of A, respectively. Based on the formula, *E*_P_ is estimated to be as high as 99.7% for (IPA@Eosin Y)/LDH with a D : A ratio of 100 : 1. To the best of our knowledge, this efficiency value is higher than those of most previously reported energy transfer systems. In order to investigate the influence of temperature on the energy transfer, the temperature-dependent spectra of the (IPA@Eosin Y)/LDH were further measured. Fig. 7c shows that the intensity of the band at 570 nm is significantly increased when the temperature was reduced from 413 to 77 K. The orange-red emission of the (IPA@Eosin Y)/LDH can also be captured before and after turning off the UV light at both 295 K and 77 K (Fig. 7d).

**Fig. 8:**
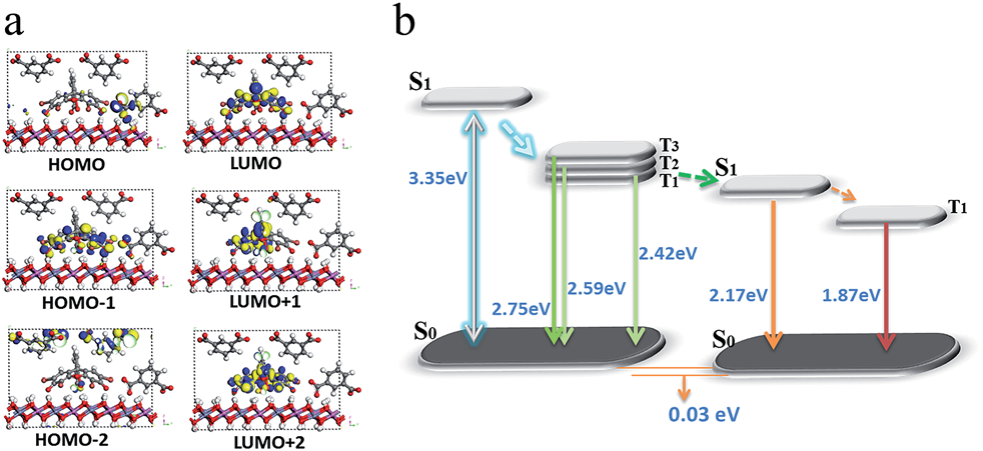
The frontier orbitals (a) and energy levels of states involved in the PET process (b) for (IPA@Eosin Y)/LDH.

To better understand the mechanism of PET between D and A within the LDH nanosheets, a TDDFT calculation was performed on an idealized model of the IPA and Eosin Y co-assembled LDH system. The optimized host–guest layered structure shows that the IPA and Eosin Y are arranged in an ordered fashion within the LDH nanogallery, with the center–center distance between Eosin Y and adjacent IPA being 0.97 nm, and their dipole orientations being regular. The structure suggests that the arrangement of D and A guests meets the spatial requirements for energy transfer. Moreover, it was observed that the three lowest photoexcitation states are mainly derived from the HOMO–*n* (*n* = 0, 1, 2) → LUMO transitions. Frontier orbital analysis (Fig. 8a) shows that the HOMO and HOMO–2 arise mainly from the sp^2^ O/C atomic orbitals in the π-conjugated IPA, while the HOMO–1 and LUMO are mainly derived from the O/C atomic orbitals in Eosin Y. This further confirms the occurrence of a PET process between IPA and Eosin Y from a theoretical point of view. In addition, the LDH monolayer with a band gap of 5.7 eV does not participate in the PET process, but plays an energy-blocking role in hindering the interactions of guest anions in adjacent interlayer galleries, and ensures the high-efficiency energy transfer between the D/A pair at the 2D confined interfaces. Based on the calculated energy levels of D and A and the experimental observations, the detailed PET process can be illustrated schematically, as shown in Fig. 8b, in which the small energy gap between the T_1_/T_2_ states of D and S_1_ state of A favors the energy transfer.

In our opinion, the D/A energy transfer within the LDH layer can be regarded as a typical Dexter process, which involves the exchange of electrons between the excited D and the A.^45^ As we knew, the Dexter mechanism has already been used to illustrate the triplet–triplet energy transfer and to produce the triplet state of some photoactive molecules.^14,46^ In this work, the Dexter-type D/A energy transfer may be promoted upon location between 2D LDH sheets. For example, the distance between D and A meets the requirement of a Dexter-type energy transfer (usually less than 1 nm); their regulated dipole orientations also facilitate the overlap of their electron wavefunctions. Moreover, this process can also be related by several factors: (1) the heavy atom effect of four Br substituents in Eosin Y could enhance the spin–orbital coupling; (2) the absorption band in the acceptor overlaps extensively with the afterglow emission from the donor; (3) the difference in energy level between D and A is relatively small (0.25 eV).

## Conclusions

In summary, a new type of high-efficiency light energy transfer system, based on a PET donor–acceptor material, has been constructed using 2D host–guest nanohybrids designed from energy, spatial and time perspectives. A pristine PET donor material showing room-temperature phosphorescence with long-afterglow emission at the second scale was first fabricated by intercalation of IPA as the guest in an LDH host. The as-obtained crystalline IPA/LDH has an ultrathin-nanosheet morphology, and also exhibits well-defined up-conversion phosphorescence and reversible temperature-responsive emission. MD and DFT calculations demonstrate that the long-afterglow properties of IPA/LDH are related to the ordered arrangement of the guest, and the formation of an H-aggregation of IPA within the confined LDH matrix. Subsequently, co-intercalation of Eosin Y as an energy acceptor together with the IPA energy donor into the galleries between the 2D LDH nanosheets, affords a highly efficient energy transfer system (*E*_P_ = 99.7%), due to the spatial and energy confinement effects of the LDH layer and the extended lifetime of the PET process, as confirmed both experimentally and theoretically. It is anticipated that the design and assembly principles based on PET at a 2D confined host–guest interface developed in this work can readily be extended to the fabrication of a wide range of other high-efficiency energy transfer systems.

## Methods

### Synthesis of PA/LDH, IPA/LDH, and TPA/LDH nanohybrids

PA/LDH, IPA/LDH, and TPA/LDH hybrids were prepared by the coprecipitation method. The matched molar ratios of Zn^2+^/Al^3+^/PA (or IPA/TPA) were 2.0 : 1.0 : 0.5. Taking the PA/Zn_2_Al-LDH as an example, 100 mL of a solution containing Zn(NO_3_)_2_·6H_2_O (5.00 mmol) and Al(NO_3_)_2_·9H_2_O (2.50 mmol) was slowly added dropwise to 100 mL of a solution containing NaOH (15.00 mmol) and PA (1.25 mmol) with vigorous agitation under a nitrogen flow. The pH value at the end of addition was adjusted to 7.0 by further addition of 2.4 mol L^–1^ NaOH solution. The reaction mixture was subsequently heated at 60 °C for 24 h, filtered, and the resulting solid washed thoroughly with deionized water and finally vacuum-dried at 50 °C for 15 h.

### Synthesis of (IPA@Eosin Y)/LDH nanohybrids

(IPA@Eosin Y)/LDH composites were prepared by the coprecipitation method. The matched molar ratios of IPA/Eosin Y were 90 : 10, 100 : 10, 95 : 5, 98 : 2 and 100 : 1. For the typical example with an IPA/Eosin Y ratio of 100 : 1, 100 mL of a solution containing Zn(NO_3_)_2_·6H_2_O (5.10 mmol) and Al(NO_3_)_2_·9H_2_O (2.55 mmol) was slowly added dropwise to 100 mL of a solution containing NaOH (15.00 mmol), IPA (1.25 mmol) and Eosin Y (0.0125 mmol) with vigorous agitation under a nitrogen flow. The pH value at the end of addition was adjusted to 7.0 by further addition of 2.4 mol L^–1^ NaOH solution. The reaction mixture was subsequently heated at 60 °C for 24 h, filtered, and the resultant solid washed thoroughly with deionized water and finally vacuum-dried at 50 °C for 15 h.

### Structural and morphology characterization

Powder XRD patterns of all compounds were collected on a Rigaku Ultima-IV automated diffraction system with Cu Kα radiation (*λ* = 1.5406 Å). Measurements were made in a 2*θ* range of 3–70° at room temperature with a step of 0.02° (2*θ*) and a counting time of 0.2 s per step. The operating power was 40 kV, 50 mA. Thermogravimetric analysis (TGA) experiments were carried out on a Perkin-Elmer Diamond SII thermal analyzer from room temperature to 1000 °C with a heating rate of 10 °C min^–1^. Elemental analysis was performed by inductively coupled plasma (ICP) atomic emission spectroscopy on a Shimadzu ICPS-7500 instrument using solutions prepared by dissolving the samples in dilute nitric acid. Carbon, hydrogen, nitrogen, and sulfur analyses were carried out using a Perkin-Elmer Elementarvario elemental analysis instrument. The morphology of the samples was investigated by using a scanning electron microscope (SEM Hitachi S-3500) equipped with an EDX attachment (EDX Oxford Instruments Isis 300), with an acceleration voltage of 20 kV. The thickness of the sample was obtained by using atomic force microscopy (AFM) software (Digital Instruments, Version 6.12). TEM images were recorded with Philips Tecnai 20 and JEOL JEM-2010 high-resolution transmission electron microscopes. The accelerating voltage was 200 kV in each case. Solid-state cross-polarization/magic angle spinning (CP/MAS) ^13^C nuclear magnetic resonance (NMR) spectra were recorded using a Bruker BioSpin AV 300 MHz at 20 μC with a 4 mm rotor spinning at 5 kHz under a static magnetic field of 9.4 T. ^13^C spectra were obtained at a frequency of 75.48 MHz with a 5 s relaxation delay and a spectral bandwidth of 350 ppm.

### Optical properties

Photoluminescence (fluorescence and phosphorescence) spectra, time-resolved luminescence decay spectra and temperature-dependent phosphorescence spectra of the samples were performed on an Edinburgh FLS980 fluorescence spectrometer. The solution UV-vis absorption spectra were collected in the range 200–700 nm on a Shimadzu U-3000 spectrophotometer, with a slit width of 1.0 nm. Phosphorescence quantum yield values at room temperature were estimated using a Teflon-lined integrating sphere (F-M101, Edinburgh, diameter: 150 mm and weight: 2 kg) in a FLS980 fluorescence spectrometer.

### Structural model and molecular dynamic (MD) simulation

An ideal LDH layer with a 8 × 6 × 1 rhombohedral supercell with *R*3*m* space group containing 32Mg atoms and 16Al atoms was built according to our previous work.^47,48^ The lattice parameters of the 2D layer are *a* = *b* = 2*d*_110_ = 3.05 Å, based on experimental results. The supercell of the octahedral layer has 48 metal atoms and 96OH groups under the condition of *α* = *β* = 90°, and the distance between adjacent metal atoms is 3.05 Å. Therefore, a supercell was constructed, with lattice parameters *a* = 36.60 Å, *b* = 18.30 Å, and the initial *c* = 12.8 Å (experimental results), *α* = *β* = *γ* = 90°. The supercell was treated as having *P*1 symmetry and all the lattice parameters were considered as independent variables in the simulation. A three-dimensional periodic boundary condition^49^ was applied to the whole system. Then, eight IPA^2–^ anions were introduced into the supercell, with their carboxylic groups vertical to the LDH layers. According to the experimental elemental analysis, 36 water molecules were inserted into the simulated supercell based on the rule that these molecules occupy the whole available interlayer space as much as possible. As a result, the formula of the simulated structure can be expressed as: Mg_32_Al_16_(OH)_96_(C_8_H_4_O_2_)_8_·36H_2_O. Then, MD calculations were performed by a classical molecular dynamic simulation method employing a modified cff91 force field.^47^ A charge equilibration (QEq) method^50^ was used to calculate the atomic charges of the layer, in which the partial charges are +0.703*e* for Mg, +1.363*e* for Al, –0.537*e* for O and +0.243*e* for H. Other forcefield parameters for the anions were referred to the cff91 forcefield.^51^ NBO analysis^52^ was employed to calculate the partial charges of IPA anions at the B3LYP/6-31G** level using the Gaussian 03 programs.^53^ In potential energy calculations, the long-range coulomb interactions between partial charges were computed by the Ewald summation technique^49^ and a “spline cutoff” method was used to calculate the van der Waals interaction. After energy minimization was applied to the initial model, MD simulations were performed in an isothermal–isobaric (NPT) ensemble with a typical thermodynamic temperature of 293 K and typical pressures of 0.1 MPa. The Andersen method^54^ and Berendsen method^55^ were used to control temperature and pressure, respectively. The total simulation time was 300 ps with a simulation time step of 1 fs. The result shows that the system reached equilibrium with lattice parameters and total potential energy fluctuating around a constant value within the first 50 ps, so the dynamic trajectories were recorded every 20 fs in the remaining 250 ps in order to analyze the ensemble average values. The simulations were performed using the Discover module in the Material Studio software package.^56^

### Periodic density functional theory (DFT) calculation

In order to reduce the computational complexity without affecting the precision, the supercell of IPA/LDH was reduced to 1/4 of that calculated by MD simulation. DFT and TDDFT methods were performed using the Dmol^3^ (ref. 57) module in the Material Studio software package.^58^ To study the electronic structure, the typical geometric configuration derived from the MD simulation results was selected as the initial model, and the water molecules were removed from the supercell. For (IPA@Eosin Y)/LDH system, the condition with an IPA/Eosin Y ratio of 3 was selected as the typical example. The configurations were optimized by the Perdew–Wang (PW91)^59^ generalized gradient approximation (GGA) method with the double numerical basis sets plus the polarization function (DNP). The core electrons of metals were treated by effective core potentials (ECP). The SCF convergence criterion was within 1.0 × 10^–5^ hartree per atom and the convergence criterion of structure optimization was 1.0 × 10^–3^ hartree per bohr. The Brillouin zone was sampled by 1 × 1 × 1 *k*-points, and test calculations reveal that the increase in *k*-points does not influence the results.
