# Supplementary material for: Layered host–guest long-afterglow ultrathin nanosheets: high-efficiency phosphorescence energy transfer at 2D confined interface
Source: Chem Sci. 2016 Sep 6;8(1):590–9. doi: 10.1039/c6sc03515a (PMC5358535; doi:10.1039/c6sc03515a)
Supplement: Supplementary file 1 [file SC-008-C6SC03515A-s001.pdf]

Electronic Supplementary Material (ESI) for Chemical Science.
This journal is © The Royal Society of Chemistry 2016

Supporting Information for

Layered host-guest long-afterglow ultrathin nanosheets: high-efficiency phosphorescence energy transfer at 2D confined interface

Rui Gao[a] and Dongpeng Yan[ab,*]

a: State Key Laboratory of Chemical Resource Engineering, Beijing University of Chemical Technology, Beijing 100029, P. R. China

b: Key Laboratory of Theoretical and Computational Photochemistry, Ministry of Education, College of Chemistry, Beijing Normal University, Beijing 100875, China

Email: yandp@buct.edu.cn; yandp@bnu.edu.cn

Contents

Table S1: Chemical compositions

Figure S1: ^{13}C MAS NMR spectra

Figure S2: TG and DTA curves

Figure S3: SEM images of PA/LDH and TPA/LDH

Figure S4: Normalized fluorescence emission spectra.

Figure S5: Normalized steady-state photoluminescence spectra excited at 320 nm in air, and oxygen, respectively

Table S2: The comperision of the phosphorescence lifetime and quantum yield values for the typical RTP materials

Table S3: Excited state lifetimes for the intercalation and co-intercalation products

Figure S6: Total and partial electronic density

Figure S7: Calculated band structure around the Fermi energy level

Figure S8: The XRD pattern samples and normalized phosphorescence emission spectra of (IPA@Eosin Y)/LDH with different ratios

Figure S9: SEM image of (IPA@Eosin Y)/LDH

Figure S10: Normalized fluorescence emission spectra and the lifetime decay profiles of Eosin Y at 295 K

Figure S11: The lifetime decay profiles of IPA/LDH in 370 nm and 450 nm.

Table S1: Chemical compositions of PA/LDH, IPA/LDH and TPA/LDH.

Sample	Final M^{2+}/M^{3+}	Elemental analysis chemical composition
2:1 (Zn:Al) PA/LDHs	1.97	$Zn_{0.663}Al_{0.337}(OH)_2\ (C_8H_4O_4)_{0.176}1.94H_2O$
2:1 (Zn:Al) IPA/LDHs	1.69	$Zn_{0.628}Al_{0.372}(OH)_2\ (C_8H_4O_4)_{0.184}0.85H_2O$
2:1 (Zn:Al) TPA/LDHs	1.61	$Zn_{0.617}Al_{0.383}(OH)_2\ (C_8H_4O_4)_{0.205}2.91H_2O$

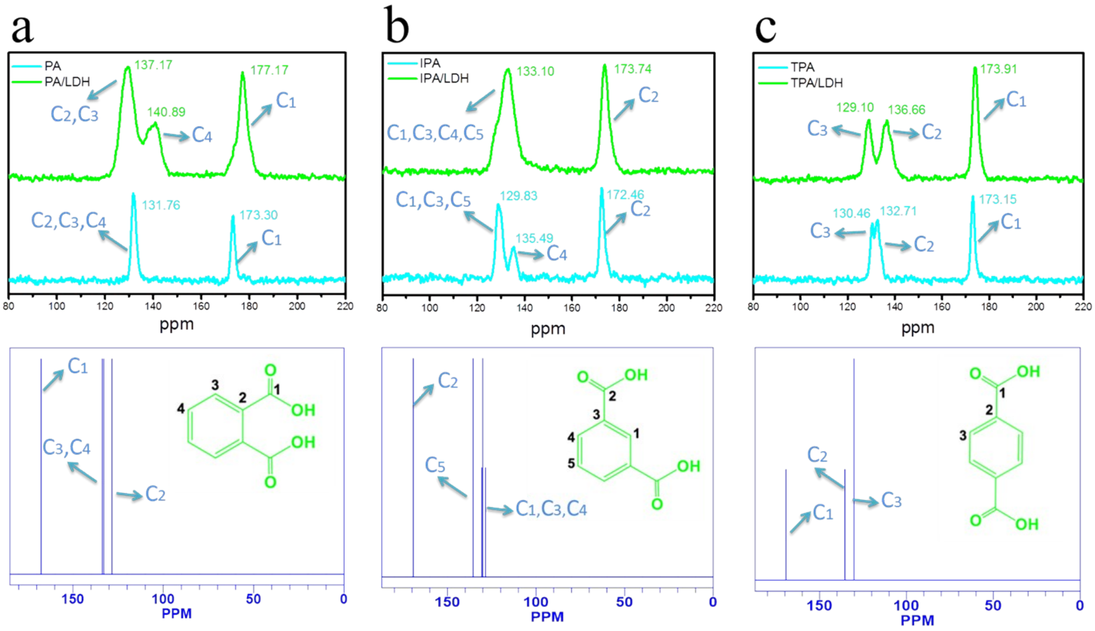

Figure S1. ^{13}C MAS NMR spectra (top: experimental; bottom: simulated based on Chem Draw 14.0 version).

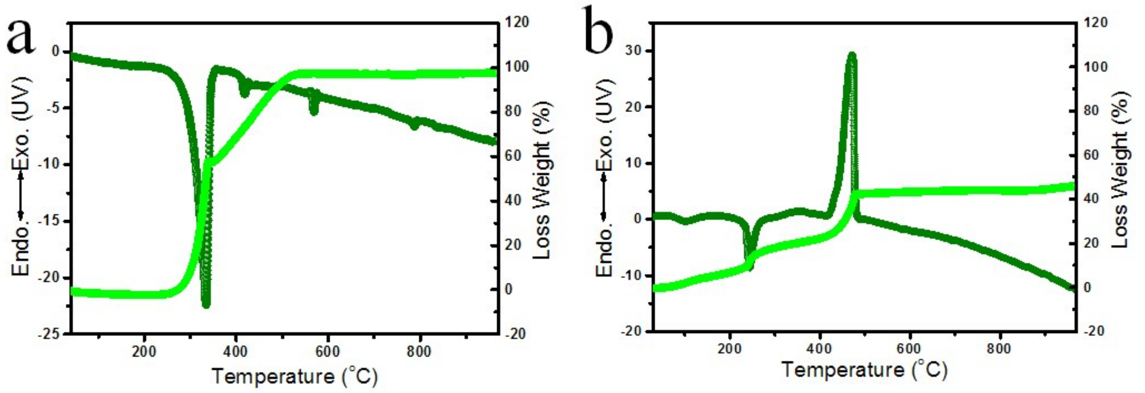

Figure S2. TG and DTA curves for (a) pure IPA, and (b) IPA/LDH.

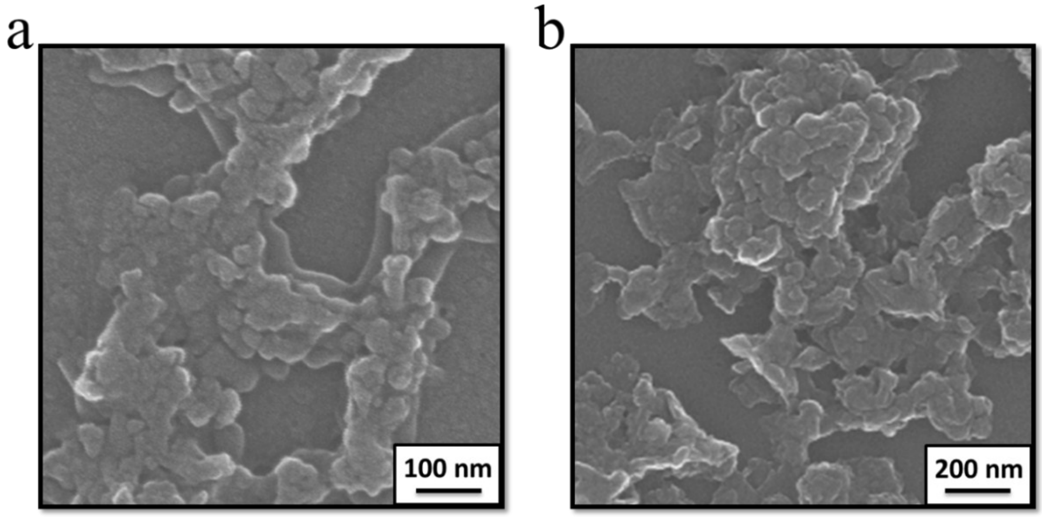

Figure S3. SEM images of (a) PA/LDH and (b) TPA/LDH.

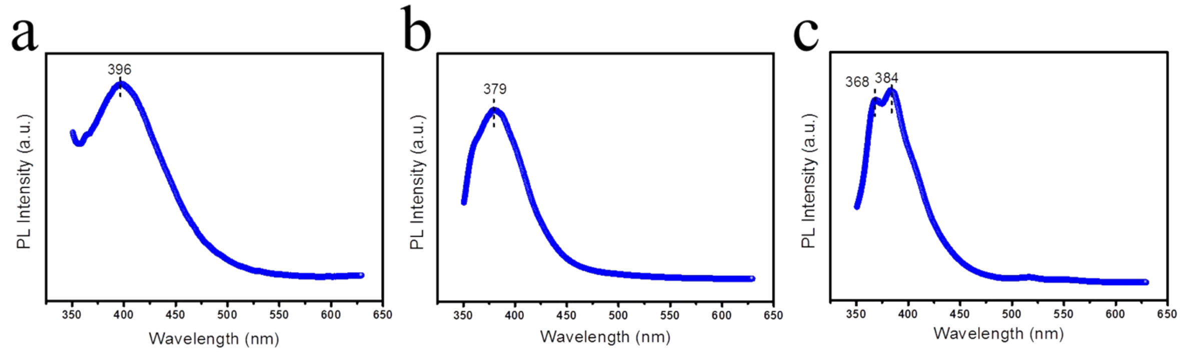

Figure S4. Normalized fluorescence emission spectra excited at 320 nm for (a) PA, (b) IPA, and (c) TPA.

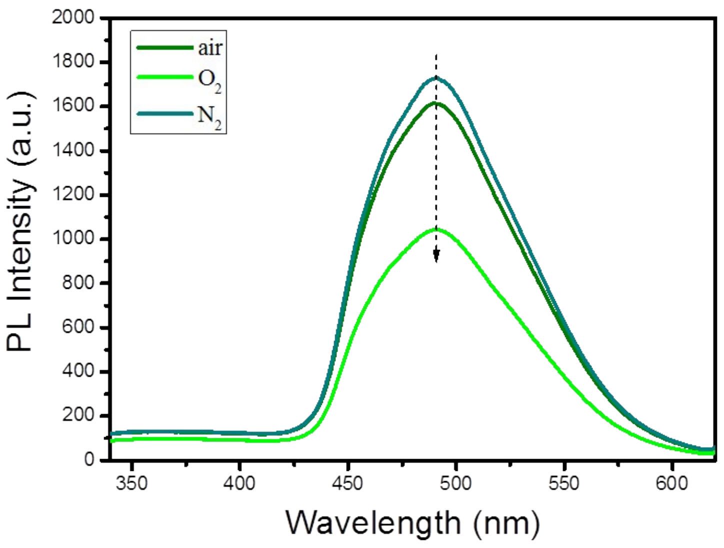

Figure S5. Normalized steady-state photoluminescence spectra of the IPA/LDH powder excited at 320 nm in air, nitrogen and oxygen, respectively.

Table S2: The comperision of the phosphorescence lifetime and quantum yield values for the typical RTP materials in refs. 30-33 and this work.

Reference number	Materials	Lifetime	Phosphorescence quantum yield
**30**	2,5-dihexyloxy-4-bromobenzaldehyde	5.4 ms	2.9 %
**30**	1 wt% mixture of 2,5-dihexyloxy-4-bromobenzaldehyde to 2,5-dihexyloxy-1,4-dibromobenzene	8.3 ms	55 %
**30**	1,4-dibromo-2,5-diheptylbenzene crystals containing 1 wt% 4-bromo-2,5-diheptylbenzaldehyde	0.1 ms	0.5 %
**30**	(2,5-dibromo-1,4-phenylene)bis(hexylsulfane) crystals containing 1 wt% 4-bromo-2,5-bis(hexylthio)benzaldehyde	6.4 ms	28 %
**30**	1,5-dibromo-2,6-bis(hexyloxy)naphthalene crystals containing 1 wt% 5-bromo-2,6-bis(hexyloxy)-1-naphthaldehyde	0.6 ms	1 %
**31**	terephthalic acid	0.5 ms	not mentioned
**31**	isophthalic acid	289.86 ms	not mentioned
**31**	tetra-fluoroterephthalic acid	17.93 ms	not mentioned

**32**	co-crystal of 1,4-diiodotetrafluorobenzene and carbazole	2.78 ms	33%
**33**	4,6-diphenyl-2-carbazolyl-1,3,5-triazine	1.06 s	1.25 %
**33**	1,4-Diethoxybenzene	710 ms	0.3 %
**33**	4,6-diethoxy-2-carbazolyl-1,3,5-triazine	1.35 s	0.6 %
**33**	2-carbazolyl-4,6-dichloro-1,3,5-triazine	490 ms	2.1 %
**33**	di(9*H*-carbazolyl)-phenylphosphine	290 ms	0.08 %
**This work**	isophthalate/LDHs	1.23 s	3.02 %
**This work**	phthalate/LDHs	0.118 s	0.71%
**This work**	terephthalate/LDHs	0.186 s	0.69%
**This work**	eosin Y/LDH	0.12 ms	2.05 %
**This work**	(isophthallate@eosin Y)/LDH	4.32 ms	2.67%

30. O. Bolton, K. Lee, H. J. Kim, K. Y. Lin, J. Kim, *Nat. Chem.,* 2011, **3**, 205.

31. Y. Gong, L. Zhao, Q. Peng, D. Fan, W. Z. Yuan, Y. Zhang, B. Z. Tang, *Chem. Sci.,* 2015, **6**, 4438.

32. H. Y. Gao, Q. J. Shen, X. R. Zhao, X. Q. Yan, X. Pang, W. J. Jin, *J. Mater. Chem.,* 2012, **22**, 5336.

33. Z. An, C. Zheng, Y. Tao, R. Chen, H. Shi, T. Chen, Z. Wang, H. Li, R. Deng, X. Liu, W. Huang, *Nat. Mater.,* 2015, **14**, 685.

Table S3: Excited state lifetimes for the intercalation and co-intercalation products monitored at different wavelengths and different temperatures.

Samples	λ_{em}	Temperature	m	τ_i	A_i (%)	$<\tau>$	χ^2
PA/LDH	450 nm	290 K	3	8.40 ms	5.57	118 ms	1.03
				48.6 ms	38.75		
				178 ms	55.67		
TPA/LDH	475 nm	290 K	2	37.4 ms	23.68	186 ms	1.07
				232 ms	76.32		
IPA/LDH	477 nm	290 K	2	0.51 s	22.08	1.23 s	1.28
				1.44 s	77.92		
IPA/LDH	370 nm	295 K	1	0.60 ns	100	0.60 ns	0.98

IPA/LDH	450 nm	295 K	2	0.10 s	46.25	262 ms	1.41
				0.40 s	53.75		
IPA/LDH	477 nm	295 K	2	0.46 s	24.98	1.08 s	1.08
				1.29 s	75.02		
IPA/LDH	450 nm	77 K	2	0.51 s	18.22	1.90 s	1.29
				2.21 s	81.78		
IPA/LDH	477 nm	77 K	2	1.80 s	16.57	3.21 s	1.33
				3.56 s	83.43		
IPA/LDH	512 nm	77 K	2	0.29 s	13.82	1.19 s	1.12
				1.38 s	86.18		
IPA/LDH	477 nm	335 K	3	8.48 ms	12.51	0.13 s	1.49
				45.8 ms	41.05		
				232 ms	46.44		
Eosin Y	560 nm	295 K	2	0.19 ms	55.12	0.12 ms	1.35
				0.03 ms	44.88		
(IPA@/ Eosin Y)/LDH	370 nm	295 K	1	0.62 ns	100	0.62 ns	1.13
(IPA@/ Eosin Y)/LDH	450 nm	295 K	2	1.52ms	42.14	0.87 ms	1.37
				0.41ms	57.86		
(IPA@/ Eosin Y)/LDH	570 nm	295 K		0.95 ms	100	0.95 ms	1.06
(IPA@/ Eosin Y)/LDH	660 nm	295 K	2	5.10ms	82.14	4.32 ms	1.22
				0.73ms	17.86		

m stands for the mono-, double- or three-exponential fitting of the fluorescence decay curve; τ_i is the excited state lifetime, A_i stands for the percentage of τ_i. The fitting goodness is indicated by the value of χ^2. In the double-exponential case, $<\tau> = A_1\tau_1 + A_2\tau_2$; $A_1 + A_2 = 1$; In the three-exponential case, $<\tau> = A_1\tau_1 + A_2\tau_2 + A_3\tau_3$; $A_1 + A_2 + A_3 = 1$.

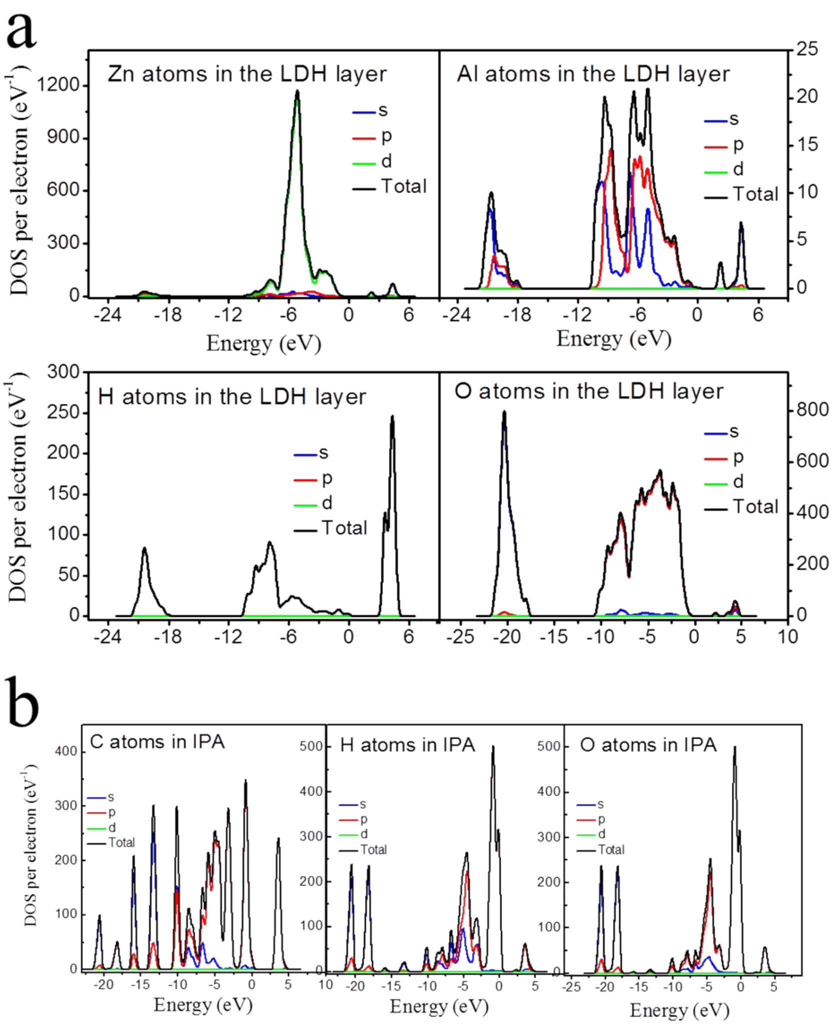

Figure S6. Total and partial electronic density of state (TDOS and PDOS) for the (a) Zn-Al/LDH layer and (b) interlayer IPA system.

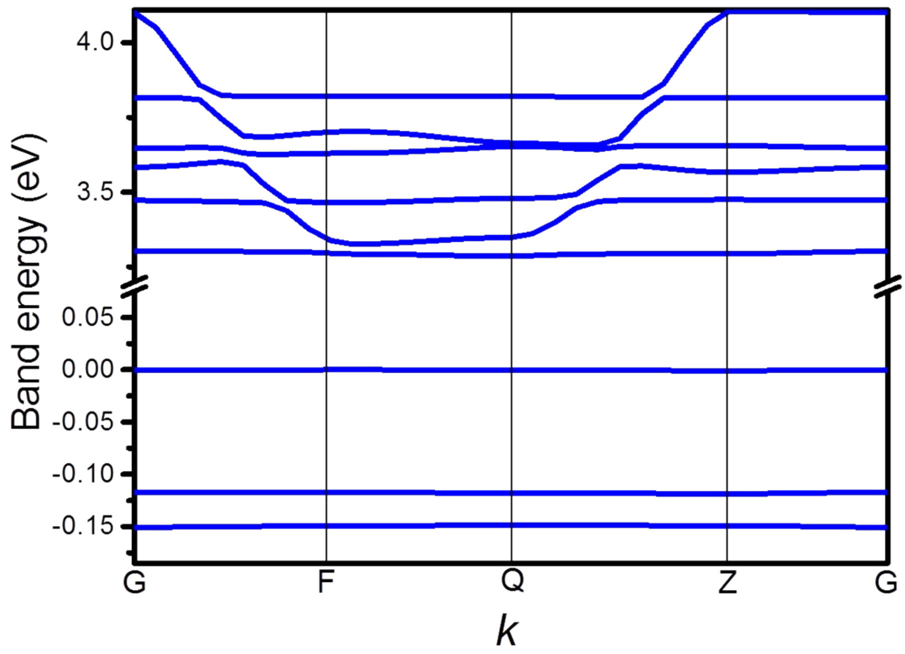

Figure S7. Calculated band structure around the Fermi energy level for the IPA/LDH system. G (0,0,0), Z (0,0,1/2), F (0,1/2,0) and Q (1/2,0,0) are the selected reciprocal points in the first Brillouin Zone (BZ).

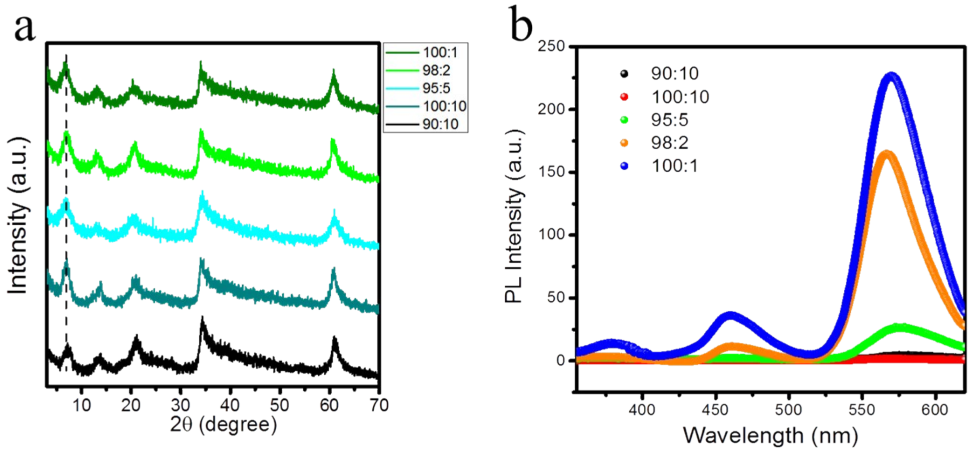

Figure S8. (a) The XRD pattern samples and (b) normalized phosphorescence emission spectra of (IPA@Eosin Y)/LDH hybrids with different ratios (90:10–100:1)

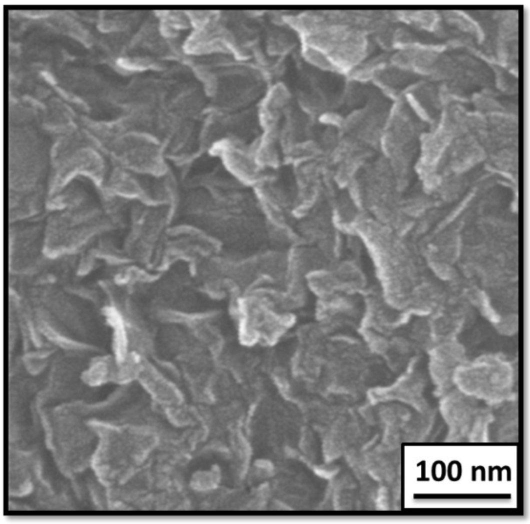

Figure S9. SEM image of (IPA@Eosin Y)/LDH.

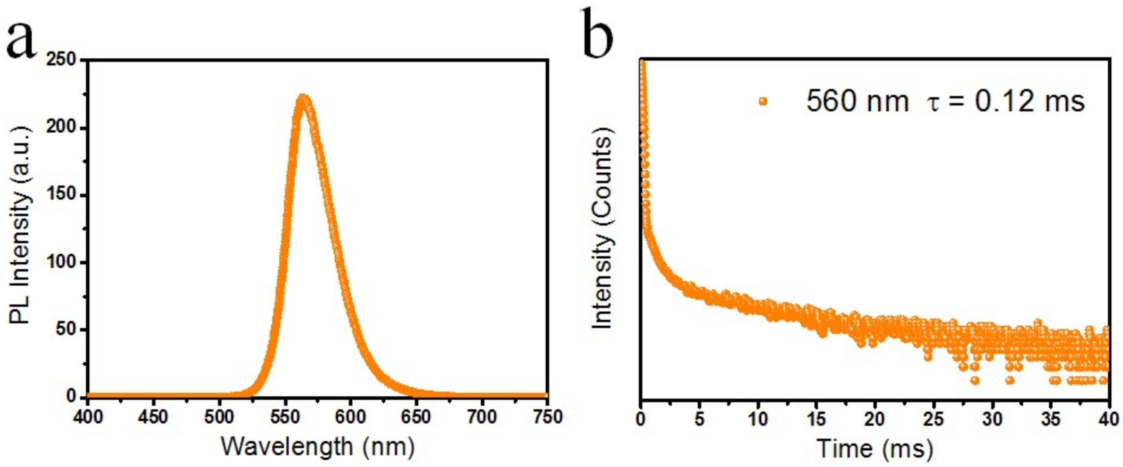

Figure S10. (a) Normalized fluorescence emission spectra of pristine Eosin Y solution (10^{-5} mol/L). (b) The lifetime decay profiles of Eosin Y solution at 295 K.

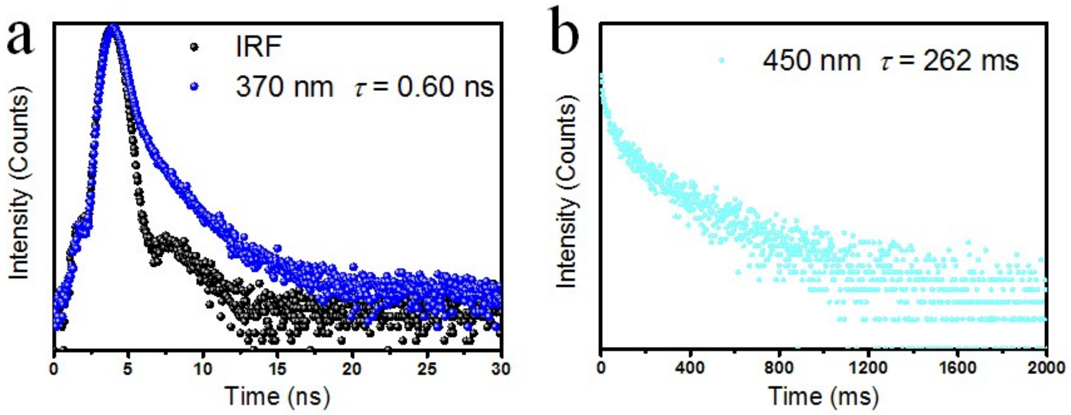

Figure S11. The lifetime decay profiles of IPA/LDH at 370 nm (a) and 450 nm (b).
